# Adult Supracardiac Total Anomalous Pulmonary Venous Connection: An Uncommon Entity

**DOI:** 10.7759/cureus.107632

**Published:** 2026-04-24

**Authors:** Sachin Talwar, Tanvi Sunil, Navnita Kisku, Sambhunath Das, Vishal V Bhende

**Affiliations:** 1 Cardiothoracic and Vascular Surgery, All India Institute of Medical Sciences, New Delhi, New Delhi, IND; 2 Cardiac Anaesthesiology and Critical Care, All India Institute of Medical Sciences, New Delhi, New Delhi, IND; 3 Pediatric Cardiac Surgery, Sri Sathya Sai Sanjeevani Centre for Child Heart Care and Training in Pediatric Cardiac Skills, Navi Mumbai, IND; 4 Pediatric Cardiac Surgery, Bhanubhai and Madhuben Patel Cardiac Centre of Shree Krishna Hospital, Bhaikaka University, Anand, IND

**Keywords:** adult congenital heart disease, anomalous pulmonary venous connection, congenital heart disease, cyanotic congenital heart disease, late presentation of congenital heart disease

## Abstract

Total anomalous pulmonary venous connection (TAPVC), a congenital cardiac disorder, usually manifests in early infancy. Its presentation may be catastrophic if the drainage site into the systemic venous circulation is obstructed. Conversely, in the absence of such obstruction, its presentation may be delayed. Very few sporadic case reports or small series describing its presentation in adult patients are available. In this report, we present an adult patient with this diagnosis, in whom all four pulmonary veins had joined to form a common pulmonary venous confluence (CPVC) that drained into a left vertical vein, which in turn drained into the innominate vein.

## Introduction

Total anomalous pulmonary venous connection (TAPVC) is an uncommon congenital cardiac anomaly in which all pulmonary veins, which normally carry oxygenated blood from the lungs to the left atrium, fail to connect to the left atrium and instead drain into the systemic venous circulation. This anomaly accounts for approximately 2% of all congenital heart diseases and results in complete mixing of oxygenated and deoxygenated blood within the systemic venous circulation [1]. In most cases, survival depends on the presence of an interatrial communication, typically an atrial septal defect (ASD), which allows oxygenated blood to reach the left heart and systemic circulation [2].

TAPVC most commonly presents during the neonatal period or early infancy and is frequently associated with significant morbidity and mortality if untreated. Mortality may reach nearly 50% within the first three months of life and up to 80% within the first year without surgical correction [2]. Early clinical deterioration is usually related to pulmonary venous obstruction, pulmonary congestion, and elevated pulmonary vascular resistance, which can rapidly lead to hemodynamic compromise [3]. Consequently, most patients are diagnosed early and undergo surgical repair during infancy.

However, in rare circumstances, TAPVC may remain undiagnosed until adolescence or adulthood. Late presentation is generally associated with unobstructed pulmonary venous drainage and the presence of a large, non-restrictive ASD that permits adequate intracardiac mixing and maintains relatively normal pulmonary vascular resistance [4-10]. These physiological conditions may allow patients to remain relatively asymptomatic for many years, delaying diagnosis until adulthood.

Adult presentation of TAPVC is therefore exceedingly rare, and most of the available literature consists of isolated case reports or small case series [4-10]. Recognition of such cases is clinically important because delayed diagnosis may still permit successful surgical correction with favorable outcomes. In this report, we describe a rare case of unobstructed supracardiac TAPVC (in which the pulmonary veins drain into systemic veins above the heart, typically via a vertical vein into the innominate vein) presenting in the third decade of life.

## Case presentation

The Institutional Ethics Committee (IEC-2) of Bhaikaka University, Anand, Gujarat, approved this study (Approval No. IEC/BU/2025/Cr.36/194/2025, dated 06/06/2025), and informed consent for publication of this report and the accompanying images was obtained from the patient.

A 25-year-old male patient presented with difficulty in breathing on moderate exertion, corresponding to New York Heart Association (NYHA) class II, for the past 2 years, and distal enlargement of the fingertips since childhood. He had no comorbidities and no prior hospital admissions. He had been taking cardiac medications (diuretics and rate-control agents) only since the diagnosis of a cardiac ailment at another hospital. On examination, the patient was thin-built, with a heart rate of 90/min, regular rhythm, and blood pressure (BP) of 100/50 mmHg. He had peripheral cyanosis and digital clubbing. Pulsation of the supraclavicular vein, best appreciated in the sitting position, was observed in addition to a fixed split second heart sound and a systolic-diastolic double murmur in the pulmonary area. The systolic murmur was moderately intense and harsh, radiating to the entire precordium, left axilla, left side of the neck, and upper back. The diastolic murmur was faint.

**Figure 1:**
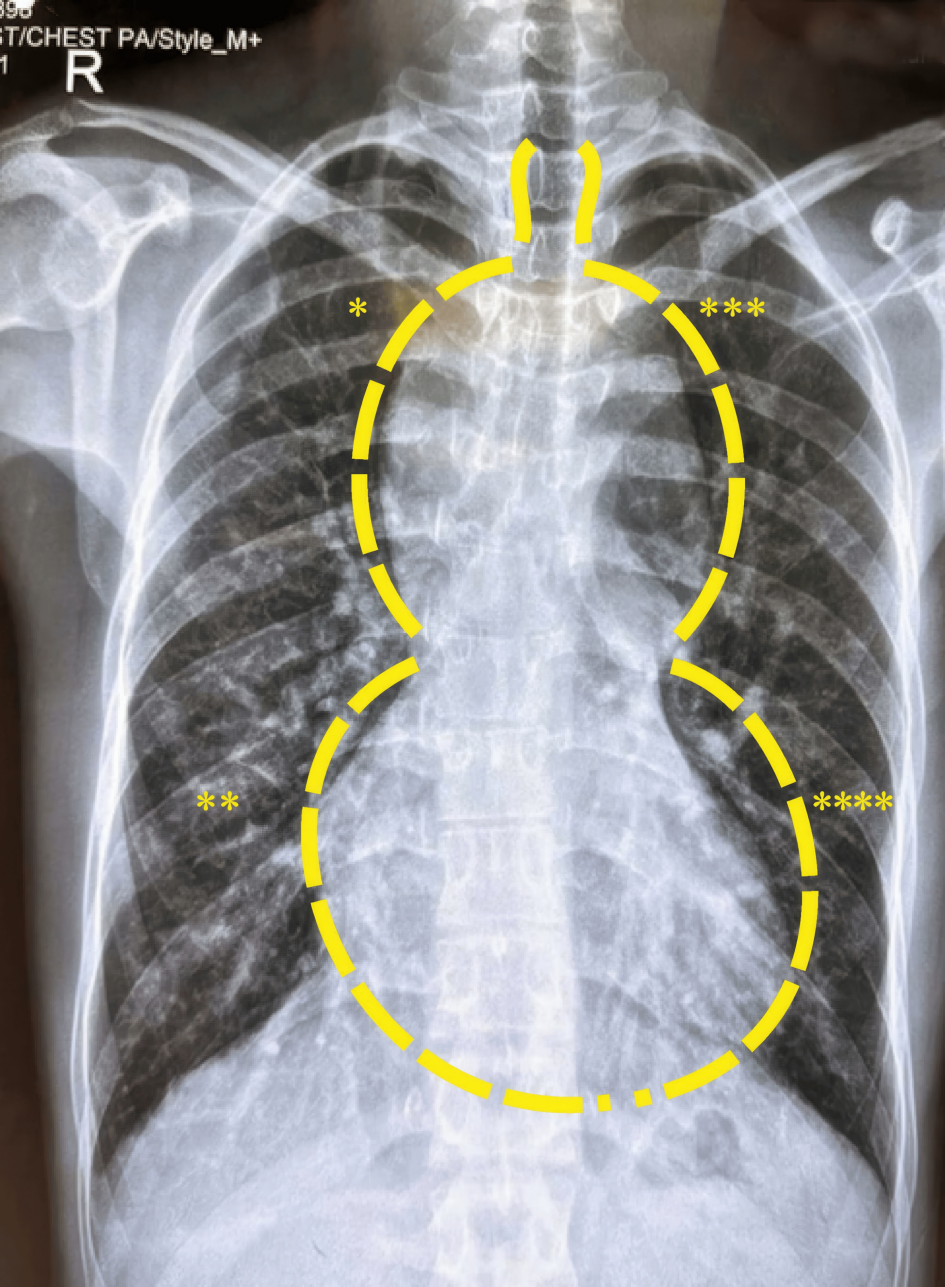
Preoperative thoracic X-ray of TAPVC. *: Dilated superior vena cava; **: Right heart border; ***: Dilated vertical vein; ****: Left heart border. TAPVC: Total anomalous pulmonary venous connection. Image credit: Dr. Sachin Talwar

The electrocardiogram showed sinus rhythm, right ventricular volume overload, and right bundle branch block. Posteroanterior chest radiography indicated increased pulmonary blood flow and a widened superior mediastinum, with features of the typical “snowman” appearance (Figure 1).

**Figure 2:**
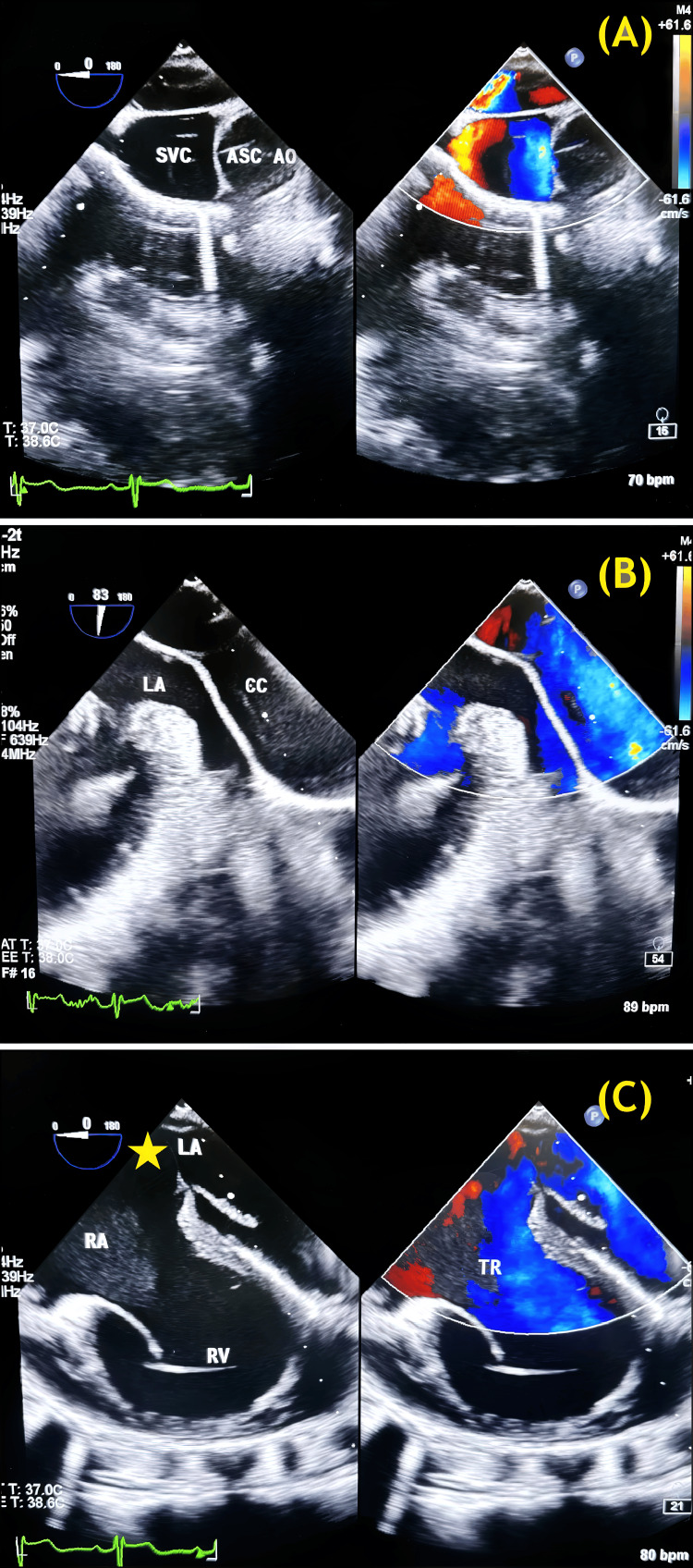
Preoperative TEE images showing (A) a dilated SVC; (B) separation between the CC and LA; and (C) the LA and RA, with a large unrestrictive ASD (starred) between them. TR is also seen. SVC: Superior vena cava; AO: Ascending aorta; CC: Common chamber; LA: Left atrium; RA: Right atrium; RV: Right ventricle; TR: Tricuspid regurgitation; ASD: Atrial septal defect; TEE: Transesophageal echocardiography. Image credit: Dr. Sambhunath Das

Echocardiographic examination showed situs solitus, levocardia, and normally related great arteries. All four pulmonary veins (PVs) formed a common chamber (CC), which drained into a left-sided vertical vein (VV), further draining into a dilated superior vena cava (SVC) through the innominate vein (IV) (Figure 2).

There was a large ostium secundum atrial septal defect (OS-ASD), nonrestrictive to flow and amounting to a common atrium, with right-to-left shunting. The right atrium (RA), right ventricle (RV), and pulmonary artery (PA) were dilated, and there was mild tricuspid regurgitation (TR) and an intact ventricular septum. The estimated right ventricular systolic pressure (RVSP) was approximately 45 mmHg.

**Figure 3:**
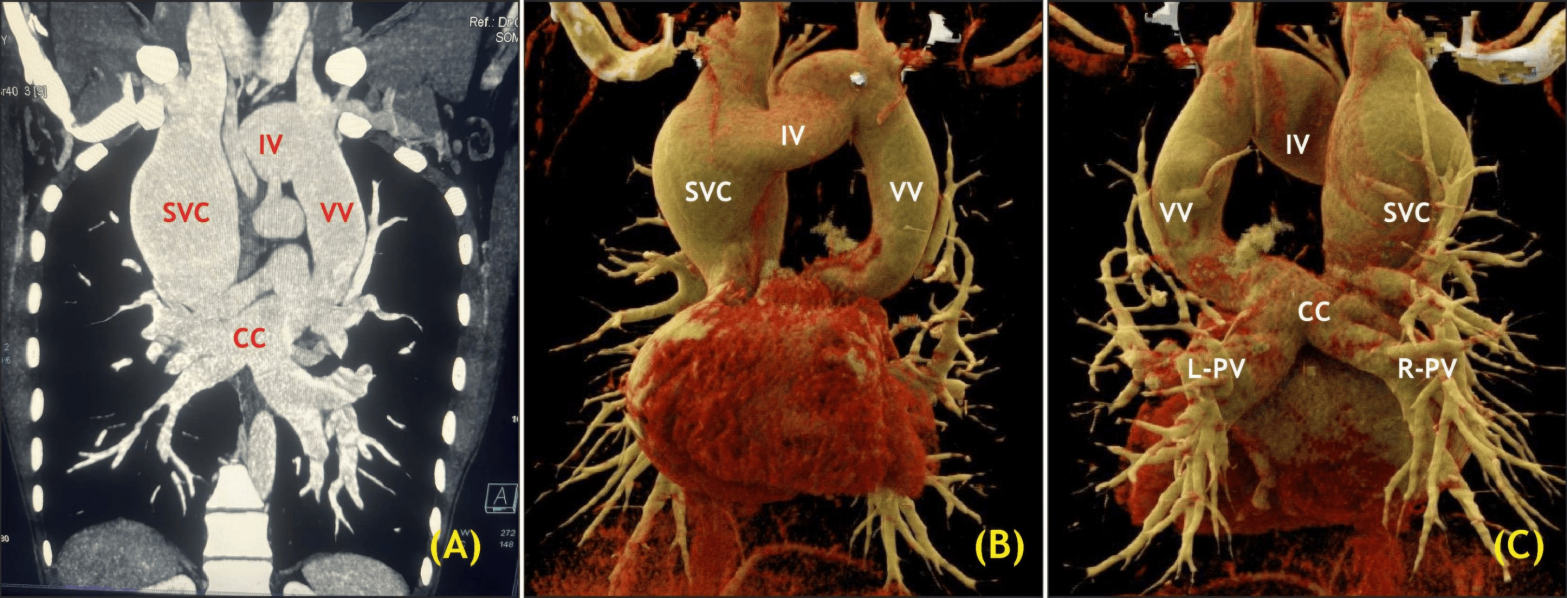
Preoperative CT angiography showing (A) the CC ascending into the VV; (B) 3D reconstruction (anterior view); and (C) 3D reconstruction (posterior view). SVC: Superior vena cava; IV: Innominate vein; CC: Common chamber; L-PV: Left pulmonary veins; R-PV: Right pulmonary veins; CT: Computed tomography. Image credit: Dr. Sambhunath Das

The small left atrium (LA) was not connected to any PV. Right and left ventricular function was normal. Cardiac CT angiography is shown in Figure 3.

CT angiography confirmed the diagnosis, showing supracardiac TAPVC with all PVs draining into the CC, then into the VV, IV, and SVC; OS-ASD; and dilatation of the RA and RV. Invasive cardiac catheterisation was not performed because the estimated PA pressures on 2D echocardiography were within normal limits.

Standard cardiopulmonary bypass was established with aorto-bicaval cannulation and systemic cooling to 28°C. The aortic cross-clamp time was 104 minutes, and the total cardiopulmonary bypass time was 207 minutes. The surgical approach was via a standard median sternotomy. The IV was pink in colour. The pericardium was opened in the midline. When the heart was lifted toward the right side, a pink chamber (CC) was seen near the LA that did not communicate with it and instead drained into the IV on the left side. Following aortic cross-clamping, a single dose of Del Nido cold blood cardioplegia was injected into the aortic root to achieve diastolic cardiac arrest. The right pleura was opened widely, and the pericardium on the right side was incised near the right phrenic nerve. The heart was then reflected into the right pleural cavity, and a posterior approach was used to anastomose the CC to the LA and left atrial appendage (LAA) using 6/0 polypropylene suture. After opening the RA, a large ASD was seen and closed with a large polytetrafluoroethylene patch, leaving a small 4 mm fenestration so that, in the event of raised PA pressures, it could serve as a “pop-off” to decompress the right side of the heart. Following this, the VV was ligated at its junction with the IV on the left side. The aortic cross-clamp was removed following standard de-airing manoeuvres, and after gradual rewarming, cardiopulmonary bypass was discontinued. Intravenous dobutamine at 10 mcg/kg/min, milrinone at 0.5 mcg/kg/min, and adrenaline at 0.05 mcg/kg/min were administered.

**Figure 4:**
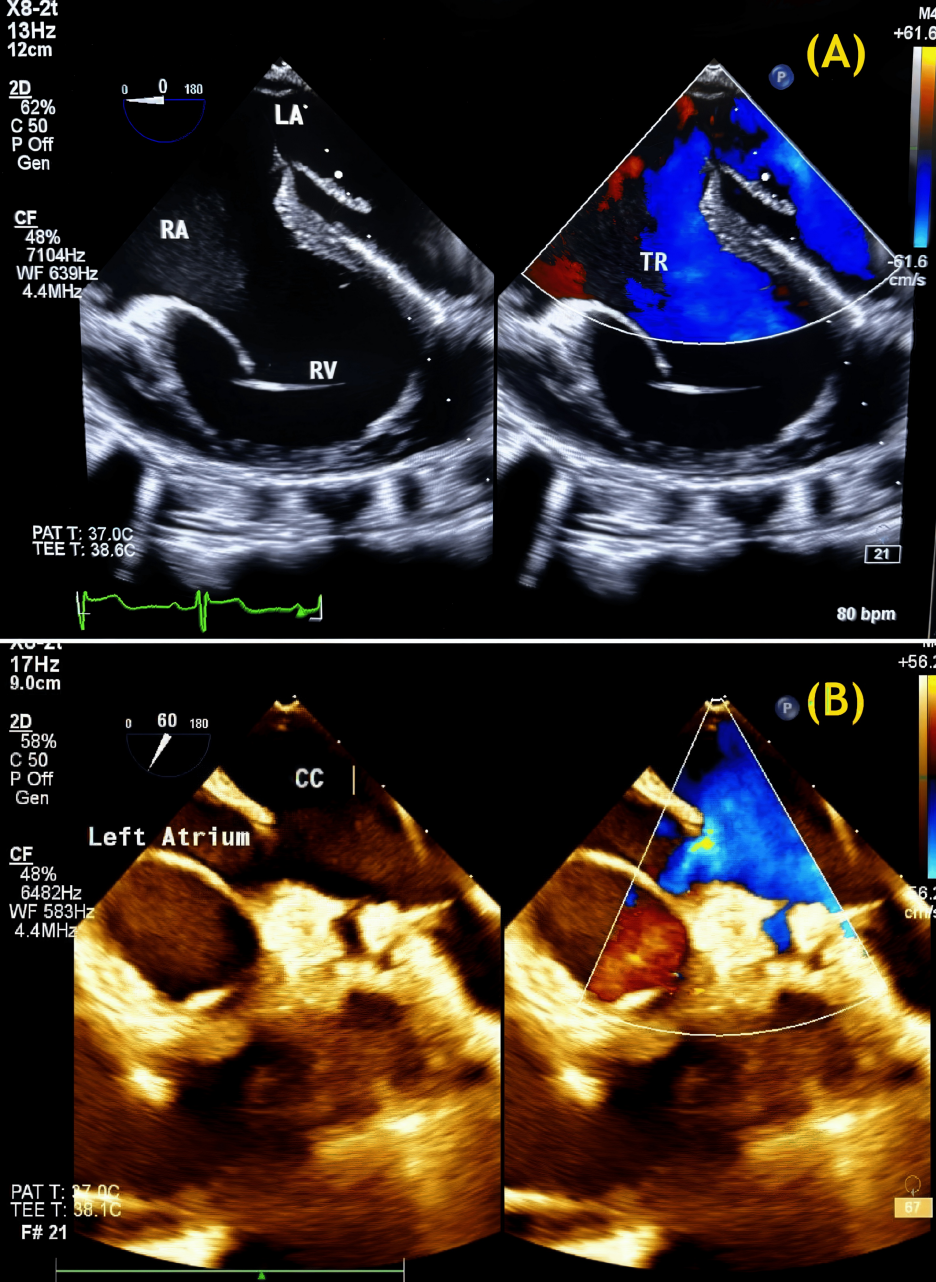
Postoperative TEE demonstrated (A) wide communication between the CC of the pulmonary veins and the LA; and (B) the connection created between the common chamber and the LA. CC: Common chamber; LA: Left atrium; TEE: Transesophageal echocardiography. Image credit: Dr. Sambhunath Das

Intraoperative, postoperative, and transoesophageal echocardiography demonstrated wide communication between the common PV chamber and the LA (Figure 4).

After transfer to the ICU, his postoperative parameters were normal, and he was gradually weaned off mechanical ventilatory support after six hours. Subsequent postoperative recovery was uneventful, and he was discharged after six days.

Detailed quantitative postoperative echocardiographic measurements and long-term follow-up data are limited; however, early postoperative imaging confirmed unobstructed flow across the anastomosis, and the patient demonstrated clinical improvement.

## Discussion

There is limited knowledge regarding the manifestations and course of unrepaired TAPVC with onset beyond childhood, and most published articles are case reports or small case series. TAPVC typically presents in the neonatal period, and survival is largely dependent on the presence of an ASD [2]. When the ASD is small, RA pressure is higher, and blood flow to the LA and cardiac output are reduced. Most frequently, because the ASD allows unrestricted pulmonary blood flow, this flow is largely determined by the pulmonary-to-systemic vascular resistance ratio and ventricular chamber compliance [11].

Early presentation is due to elevated pulmonary vascular pressures and pulmonary congestion, which occur because of resistance at any level affecting flow, i.e. at the ASD or at the level of the vertical vein [12]. The majority of patients with TAPVC have severe symptoms, with some presenting in extremis, and only 20% survive the first year of life [13]. Late presentation is possible when a large, non-restrictive ASD is present, resulting in near-normal pulmonary vascular resistance [8]. A non-restrictive ASD impedes the early development of severe pulmonary artery hypertension [14] and, in the absence of pulmonary venous obstruction, such patients may survive beyond infancy. In a previously published series of 27 adult TAPVC patients from our institution, most cases demonstrated unobstructed supracardiac drainage with large, non-restrictive ASDs permitting survival into adulthood [14]. The present case aligns with these observations but is notable for its well-defined common pulmonary venous confluence (CPVC) draining via a prominent vertical vein into the innominate vein, with preserved hemodynamics and delayed symptom onset into the third decade. This case underscores the variability in anatomical and physiological adaptation in adult TAPVC and highlights that favorable surgical outcomes can be achieved even with late presentation. Furthermore, it illustrates the utility of cardiac CT angiography as a comprehensive, non-invasive modality for anatomical delineation, which in this case obviated the need for invasive catheterization in the presence of normal estimated pulmonary artery pressures. The various types of TAPVC and their corresponding surgical approaches are summarized in Table 1 [13,15-20].

**Table 1:** Summary of TAPVC types and their corresponding surgical approaches. TAPVC: Total anomalous pulmonary venous connection; ePTFE: Expanded polytetrafluoroethylene; PV: Pulmonary veins; VV: Vertical vein.

Sr. No.	Author & Year	TAPVC Type	Description	Drainage Location	Surgical Approach
1	Van Praagh R, et al. (1972) [13]	Cardiac	PVs joined directly to the heart	Coronary sinus or RA	Intracardiac
2	Tucker BL, et al. (1976) [15]	Supracardiac	PVs joined the systemic circulation above the heart	Left innominate vein, superior vena cava, or azygos vein	Superior
3	Williams GR, et al. (1964) [16]	Infracardiac	PVs joined the systemic circulation below the heart	Portal vein, hepatic vein, or inferior vena cava	Posterior
4	Chowdhury UK, et al. (2008) [17]	Mixed	Combination of different drainage sites	Variations can include any combination of the above	Combination of both
5	Shumacker HB and King H (1961) [18]	All varieties of TAPVC	Combination of different drainage sites	Variations can include any combination of the above	Biatrial
6	Lacour-Gayet F et al. (1996) [19]	Infracardiac	The sutureless method was first presented to treat PV obstruction (PVO) and was then suggested for primary treatment of TAPVC	Leaving the VV in place is preferred and it is always left open	Primary sutureless repair
7	Sarmast H, Takriti A (2019) [20]	TAPVC with pulmonary venous obstruction	A 4-day-old neonate with TAPVC accompanied by pulmonary venous obstruction	Color Doppler and 2D echocardiography demonstrated supracardiac TAPVC, concomitant with a noticeable gradient between the draining point of the VV into the left brachiocephalic vein and the PV, with acceleration > 3.0 m/sec (PV obstruction)	A new palliative technique in which an anastomosis was established without cardiopulmonary bypass (CPB) between the pulmonary venous confluence and the left atrial appendage, using Gore-Tex (ePTFE)

## Conclusions

Our patient had a large, non-restrictive ASD amounting to a common atrium and was therefore asymptomatic until adulthood. The vertical vein was also non-restrictive, and adequate mixing was present. These factors delayed the onset of pulmonary venous and arterial hypertension. They served as protective factors, resulting in late presentation and also allowing the patient to have an uneventful postoperative course.
